# Retrospective Assessment of Complementary Liquid Biopsy on Tissue Single-Gene Testing for Tumor Genotyping in Advanced NSCLC

**DOI:** 10.3390/curroncol30010045

**Published:** 2023-01-01

**Authors:** Patrice Desmeules, Matthieu Dusselier, Cédrik Bouffard, Josée Bafaro, Marc Fortin, Catherine Labbé, Philippe Joubert

**Affiliations:** 1 Service of Anatomic Pathology, Institut Universitaire de Cardiologie et de Pneumologie de Québec, Université Laval, Québec City, QC G1V 4G5, Canada; 2 Research Center, Institut Universitaire de Cardiologie et de Pneumologie de Québec, Université Laval, Québec City, QC G1V 4G5, Canada; 3 Service of Respirology, Institut Universitaire de Cardiologie et de Pneumologie de Québec, Université Laval, Québec City, QC G1V 4G5, Canada

**Keywords:** non-small cell lung cancer, liquid biopsy, biomarkers

## Abstract

Biomarker testing is key for non-small cell lung cancer (NSCLC) management and plasma based next-generation sequencing (NGS) is increasingly characterized as a non-invasive alternative. This study aimed to evaluate the value of complementary circulating tumor DNA (ctDNA) NGS on tissue single-gene testing (SGT). Ninety-one advanced stage NSCLC patients with tumor genotyping by tissue SGT (3 genes) followed by ctDNA (38 genes amplicon panel) were included. ctDNA was positive in 47% (*n* = 43) and identified a targetable biomarker in 19 patients (21%). The likelihood of positivity on ctDNA was higher if patients had extra-thoracic disease (59%) or were not under active treatment (59%). When compared to SGT, ctDNA provided additional information in 41% but missed a known alteration in 8%. Therapeutic change for targeted therapy based on ctDNA occurred in five patients (5%), while seven patients with missed alterations on ctDNA had *EGFR* mutations or *ALK* fusions. The median turnaround time of ctDNA was 10 days (range 6–25), shorter (*p* = 0.002) than the cumulative delays for the tissue testing trajectory until biomarker availability (13 d; range 7–1737). Overall, the results from this study recapitulate the potential and limitations of ctDNA when used complementarily to tissue testing with limited biomarker coverage.

## 1. Introduction

The recent advances in precision medicine have remodeled the approach for clinical management of advanced stage non-small cell lung carcinoma (NSCLC), more specifically for lung adenocarcinoma. The list of driver alterations paired with small molecule inhibitors has been expanding continuously and mandates evaluation of several biomarkers to guide patient management [1,2]. Consistently, molecular testing is evolving toward wider adoption of multigene panel testing, largely due to the enlarging access to NGS technologies in clinical laboratories. However, access to comprehensive molecular profiling for NSCLC remains unequal across regions and is also sometimes limited by insufficient tissue samples and long turn-around times (TAT) [3].

Molecular assays have been traditionally designed to work on formalin-fixed paraffin-embedded (FFPE) tissue. However, the technological advances have resulted into the capacity to perform molecular profiling directly from the circulating tumoral (ct) nucleic acids extracted from plasma, also named liquid biopsy. This non-invasive approach is a promising tool for diagnosis and monitoring of NSCLC [4]. Advantages over tissue testing include shorter TAT, risks and costs reduction inherent to procedures for acquisition of diagnostic material and the potential to capture tumor heterogeneity from multiple anatomic sites. However, the clinical sensitivity of liquid biopsy and high costs are still amongst factors slowing the adoption of this approach across the world [3].

Several studies have compared the performance of plasma versus tissue-based NGS assays [5,6,7,8,9]. Despite improvements in the availability of tissue-based NGS, comparison with minimal single-gene testing remains relevant as more than 30% of laboratories still use single assays to evaluate biomarkers in NSCLC [3]. This study aimed to review the results from a retrospective cohort of NSCLC patients who had complementary liquid biopsy testing from an access program launched during the COVID-19 pandemic. The objective was to evaluate the value of plasma NGS testing with a small DNA amplicon panel over minimal single-gene tissue testing as proposed by the last IASCLC/CAP/AMP guidelines [1].

## 2. Materials and Methods

This retrospective single center study includes patients with advanced stage (IIIB-IV) NSCLC treated and followed at the Institut Universitaire de Cardiologie et de Pneumologie de Québec-Université Laval (IUCPQ-UL, Quebec, QC, Canada) between December 2020 and February 2022, who underwent complementary liquid biopsy. Patients with metastatic or recurrent NSCLC were offered molecular testing on circulating tumor DNA (ctDNA) through a free and unrestricted access program for advanced stage cancer in Canada, the ACTT project (Access to Cancer Testing and Treatment).

Blood was collected in Streck tubes and sent immediately via a tiered shipping service in pre-packaged kits (Genolife; Quebec, QC, Canada) for ctDNA sequencing with the Follow It^®^ assay at Imagia Canexia Health (laboratory in Vancouver, British Columbia; headquarter in Montréal, Québec, Canada). This is a 38 genes amplicon-based panel covering 26 exons and 337 hotspot mutations in key genes relevant to solid tumors, enabling identification of single nucleotide variants (SNV), insertions and deletions up to 24 base pair length (INDEL) as well as copy number variations (CNV) (complete details provided on the vendor website: https://imagiacanexiahealth.com/solution/plasma-follow-it/; accessed on 26 December 2022). Regarding specifically the clinically actionable genes for NSCLC, this panel covers activating mutations in *EGFR* (exons 18 to 21), *BRAF* (exon 15), *ERBB2* (exon 20 and S310), *KRAS* (exons 2–4); *MET* coverage includes Y1253, exons 13, 14 + 25, 14–50, 14, 18; *ALK*, *ROS1* and *RET* includes key acquired resistance variants in tyrosine kinase domain, but the assay does not detect gene fusions or isoforms. Results for SNVs and INDELs are reported when variant allele fraction is equal or greater than 0,7% and 5%, respectively. The minimal acceptance criteria from the vendor are a coverage ≥500 as well as base quality and mapping quality scores of ≥30 each.

All patients also had conventional tissue biomarkers testing during their disease, which was performed at the IUCPQ-UL pathology laboratory. Procedures to obtain tissue biomarkers and liquid biopsy were not always performed in the same period or sequence in the care of the patient. Single-gene testing included PCR assay for *EGFR* activating mutations (RGQ PCR kit covering 29 variants in exons 18 to 21; Qiagen, Toronto, ON, Canada), and immunohistochemistry for fusion in *ALK* (clone 5A4; Biocare, Markam, ON, Canada) and *ROS1* (clone D4D6; CST, Danvers, NH, USA) on a Dako Autostainer (Agilent, Mississauga, ON, Canada), followed by FISH (SureFISH, Agilent, Mississauga, ON, Canada) when appropriate, as well as PD-L1 immunohistochemistry using the Dako 22C3 assay (Agilent, Mississauga, ON, Canada). A subset of patients had also complementary *BRAF* V600x PCR testing (Biocartis Idylla, Mechelen, Belgium) or NGS testing with a targeted lung cancer 17 genes panel (Archer Fusion Plex lung; Invitae, San Francisco, CA, USA) [10].

Patient’s medical records were reviewed to collect clinical, radiological and pathologic data. Response assessment was categorized as per RECIST criteria [11]. Reflex biomarker testing is not used in our center and the clinician place a request when clinically appropriated. Key dates (date of request of the procedure to obtain tissue for diagnosis; dates of specimen accessioning and pathology report release; dates of molecular pathology accessioning and biomarkers unified report release) were retrieved to estimate the turnaround time (TAT) of the entire trajectory length from first clinical visit to date of availability of biomarker results. The results were calculated for the entire subset and after excluding cases from resection specimens and where diagnosis and biomarkers were separated in time for more than an arbitrary cut-off of 30 d, aimed to reflect recurrent disease or testing retrospective material at progression. The liquid biopsy results were classified as informative if any mutation was identified, either a known oncogenic driver (known recurrent hot-spot activating mutations in genes of the MAPK/ERK pathway or oncogenic fusions) or a passenger alteration, or uninformative (clinically) if no alteration was identified (negative for any variant with satisfactory quality metrics). All liquid biopsy testing reports included in this study met the vendor’s quality metrics. Candidate targetable driver alterations were defined based on key alterations included in the most recent NCCN guidelines [2].

Statistical analyses (Student’s *t*-test and chi-square test) were performed using GraphPad Prism, version 9.1.0 (GraphPad Software, San Diego, CA, USA) and a 5% cut-off for statistical significance.

## 3. Results

A total of 91 patients were included in this analysis. Patient’s clinical characteristics are shown in Table 1. The study population was characterized by a slight predominance of female (59%) and a marked predominance of stage IV (92%) and non-squamous histology (98%); two patients with squamous cell carcinoma and atypical clinical presentation for which clinicians had requested biomarker testing beyond PD-L1 were included. At the time of ctDNA testing, most patients (63%; *n* = 58) had completed at least one line of treatment and 56% (*n* = 51) had extra-thoracic disease. All patients had at least a known *EGFR* and *ALK* status, but only 84% had the complete *EGFR/ALK/ROS1/PDL1* assessment combination, mainly due to testing performed prior to the study dates and local regulatory approval of the assays for *ROS1* and *PD-L1* testing. This cohort included only 13 patients (14%) with known actionable driver mutation at time of ctDNA testing based on single-gene testing.

**Figure 1:**
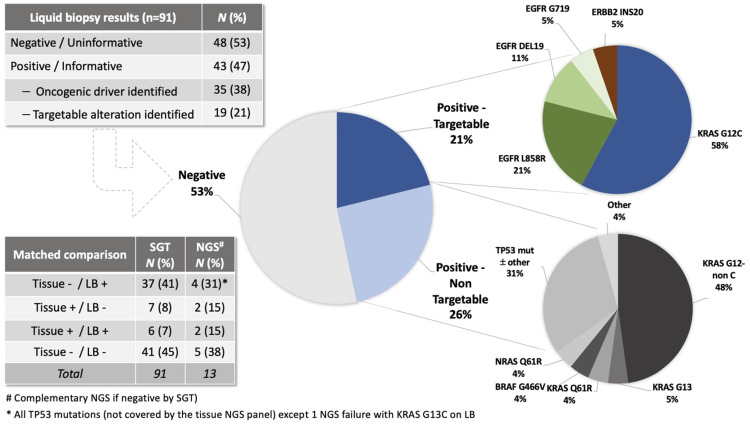
Summary of liquid biopsy (LB) results in the cohort with breakdown of molecular findings and comparison of the biomarker outcome with matched tissue evaluation by single-gene testing (SGT). NGS: next-generation sequencing with a 17 genes panel covering all guideline-recommended biomarkers.

**Figure 2:**
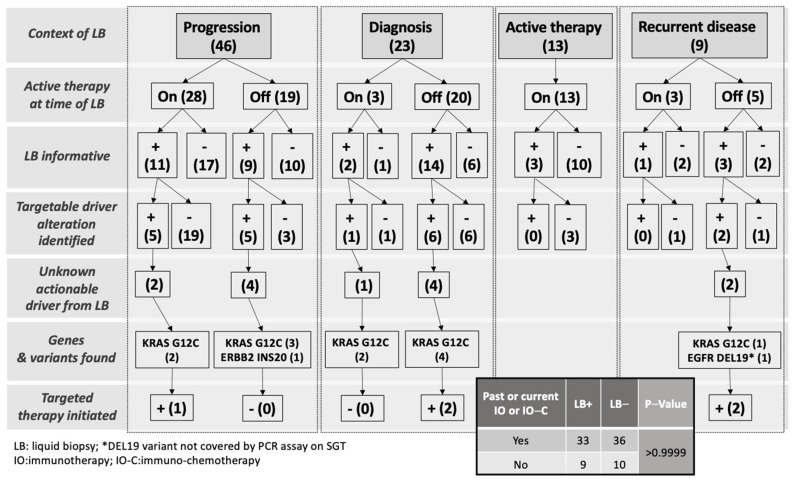
Detailed breakdown of liquid biopsy results with outcome on targeted therapy during the study. Numbers in brackets represent number of cases (*N*).

Overall, ctDNA testing was informative in 43 patients (47%), allowing for the identification of driver oncogenic alterations in 35 cases (38%) and candidate targetable alteration in 19 cases (21%); (Figure 1 and Table 2). Amongst the liquid biopsy positive cases with non-actionable alterations, *KRAS* non-G12C and *TP53* mutations were the most frequently identified (Figure 1). When compared to tissue single-gene testing results, liquid biopsy NGS panel provided additional or no additional molecular information in 37 patients (41%) and 7 patients (7%), respectively. Liquid biopsy was negative for a known molecular alteration from tissue testing in 7 patients (8%), while 45% of cases (*n* = 41) were negative by both approaches (Figure 1 and Figure 2). The distribution of *PD-L1* scores were similar in the different categories of liquid biopsy outcome (Table 2). Sub-groups analysis showed that the detection rate of liquid biopsy was higher when patients had extra-thoracic disease (59% vs. 31%; *p* = 0.0151) or were not receiving active treatment at blood draw (off-treatment or treatment naïve; 59% vs. 35%; *p* = 0.0340); Table 2.

The clinical impact of liquid biopsy testing on this cohort was further evaluated to determine the potential change in therapeutic orientation (Figure 2 and Table 3). While liquid biopsy was frequently informative, the yield of candidate targetable alterations unknown from tissue testing was relatively small and resulted in only five patients switching to targetable therapy overall (Figure 2). Four of those patients had a *KRAS* G12C mutation (*KRAS* not tested on tissue) and were subsequently offered a specific *KRAS* inhibitor while one patient had an *EGFR* deletion of 19 mutation (undetected by the tissue PCR assay) and had treatment changed to an *EGFR* tyrosine kinase inhibitor. One patient with *ERBB2* INS20 could not be offered targeted therapy before dying of disease. On the other hand, seven cases had driver alterations identified on tissue testing undetected on liquid biopsy. All these patients had targetable alterations, including five activating mutations in *EGFR* and two *ALK* fusions (Table 4). The distribution of patients who received either a previous or current therapeutic line including checkpoint-inhibitor (ICI) or ICI-chemotherapy combination in this cohort was not different in the categories of liquid biopsy result (Figure 2 insert).

**Figure 3:**
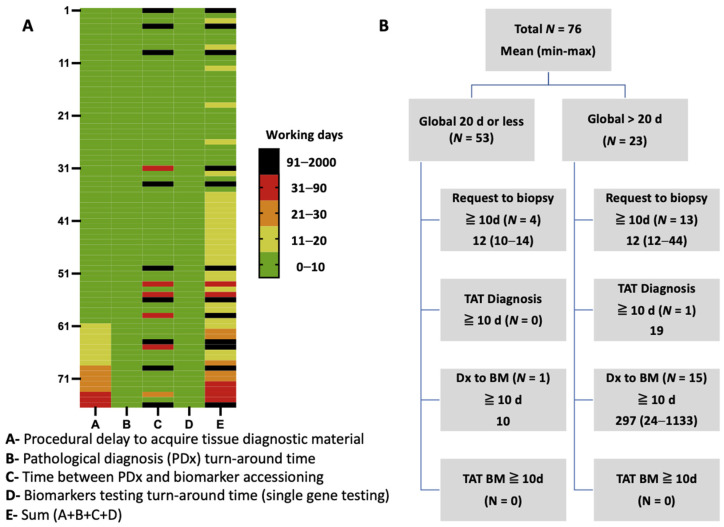
(**A**) Heat-map of turn-around time (TAT) to complete biomarker testing by tissue single-gene testing per main trajectory category (working days). (**B**) Breakdown of samples with full trajectory cumulative turnaround time divided with a threshold of 20 days.

Even though this study was not designed to compare the TAT of matched tissue and liquid biopsy testing, as they were not concomitant, an indirect comparison was possible. For the 91 samples sent for liquid biopsy testing, TAT from blood draw to result was 10 working d on average (median 10 d; range 6–25 d) (Table 5). Complete date retrieval for tissue biopsy trajectory timelapse was possible for a subset of 76 cases. Tissue pathological diagnosis and biomarker testing TAT were fast in this subgroup (mean of 2.3 and 2.8 d, respectively, median 2 d each), as the pre-analytical delay between the clinical request and completion of procedures to acquire diagnostic material (7.9 d on average; median 4 d; range 1 to 4). Biomarker testing was often requested at time of progression, then long after the initial diagnosis, as reflected by the long interval between diagnosis and biomarker request dates on average (60.8 d; median 2 d) (Table 5). The cumulative delay to obtain biomarker results on tissue was on average 73.7 d (median 13 d), decreasing to 14.4 d (median 12 d) when excluding retrospective requests over 30 d and past resection specimens. Both scenarios were significantly longer in comparison to the liquid biopsy TAT observed in this cohort (t = 3.1136, *p* = 0.002 and t = 4.086, *p* < 0.0001, respectively). Figure 3A illustrates the delays for the four main steps in patient’s trajectory from clinical visit to biomarker availability for treatment decision making. Overall, 53 cases (70%) were within 20 working days by tissue single-gene testing, and for those exceeding this cut-off (*n* = 23; 30%), longer delays between tissue request and biopsy or between diagnosis and biomarker request were the most frequently seen (Figure 3B).

## 4. Discussion

The results of this retrospective cohort analysis offer a real-life perspective about the yield and impact of integrating a plasma-based ctDNA NGS targeted assay in advanced stage NSCLC care. It provides insight about the expected positivity rate of liquid biopsy NGS in comparison with tissue single-gene testing, while exposing some clinical factors potentially associated with a higher likelihood of positivity. It also provides an estimate of the potential clinical impact of liquid biopsy when compared to biomarker testing with conventional methods.

The rate of informative cases on liquid biopsy (47%) recapitulates one key factor rendering clinically attractive a plasma-based approach in NSCLC genotyping. Indeed, liquid biopsy provided a high likelihood of capturing molecular information useful for patient management in 10 d on average. However, this clinical sensitivity rate of liquid biopsy is slightly inferior compared to other studies with similar advanced stage NSCLC populations, where it often exceeded 60% [12,13,14]. It is also lower from what would be expected by using tissue NGS with similar targets coverage in the same population, with a high prevalence of Caucasian, smokers and *KRAS* mutations. Direct inter-study and inter-population comparisons remain difficult and imperfect due to the high level of complexity and variability of the assays involved, notably the size and content of panels, as well as the pre-analytical factors. While the number of genes and type of alterations captured are important, it is uncertain whether the inability to detect gene fusions or isoforms (*ALK*, *ROS1*, *RET* and *METex14)* significantly influenced the rate of detection in this study, due to the relative rarity of fusions. Per example, some higher rate of positivity from liquid biopsy NGS were reported using a larger panel also lacking fusion capture [14]. Nonetheless, plasma-only testing using such assay could not entirely replace tissue testing since minimal requirement for NSCLC would not be met (missing *ALK* and *ROS1* fusions).

In addition, inequivalent molecular testing strategies precluded determination of the formal analytical sensitivity in this study (liquid biopsy NGS compared to tissue single-gene testing). Concordance was estimated to be 71% using the same ctDNA panel for mutations [15]. Overall, genomic profiling on tissue is expected to have a higher yield than plasma regarding guideline-recommended biomarkers [9]. Moreover, complex clinical factors are likely determinants of the success of a plasma-based assay. This is reflected in some findings here reproducing previous observations where a greater liquid biopsy positivity likelihood was seen when disease had spread outside the thorax [16] or was not actively treated [12,17]. Maybe vascular dissemination associated with distant metastasis and absence of tumor control by therapeutic agents are factors facilitating tumor DNA release, but more research is needed to better understand factors associated with ctDNA shedding and the effects of active therapy on it.

Beyond the diagnostic yield of liquid biopsy observed in this study, the clinical impact of the molecular data obtained was also evaluated. Genotyping information was acquired undoubtedly more often in plasma ctDNA NGS than in tissue single-gene testing (46% vs. 18% of patients, respectively), even if the panel did not include all guideline-recommended alterations. Despite this additional information from plasma genotyping, translation into therapeutic change for druggable oncogenic drivers was relatively modest in this cohort. Indeed, out of 91 patients, only 11 patients had unknown potentially actionable alterations and 5 patients ultimately received matched targeted therapy. This low yield must be contextualized considering the local regulatory environment at time of the study, where access to therapeutic agents associated with biomarkers outside of currently approved and reimbursed indications (limited to *EGFR*, *ALK* and *ROS1*) is challenging. The observation that only five out of nine patients with *KRAS* G12C and one patient with *ERBB2* INS20 did not receive matched therapy likely reflects this reality. In addition, it is important to remind that a large part of NSCLC management in this cohort was driven by tissue *PD-L1* status. While patients with the highest *PD-L1* level of expression show the most benefit, a large proportion of NSCLC patients now receive immune checkpoint immunotherapy (ICI) at some point during their treatment, alone or in combination with chemotherapy, as recapitulated in this cohort. The regulatory acceptance context facilitating access and global positive clinical effects and tolerability of ICI might have played a role in some cases to defer a therapeutic change toward any drug out of approved indications with hypothetical benefit.

In parallel, two out of seven actionable alterations found only by tissue testing in this cohort were *ALK* fusions. Similar discrepancies with clinically relevant fusions involving *ALK* and *ROS1* as well as *MET*ex14 isoform were noted in other comparative studies between liquid and tissue NGS. This was described using either a hybrid-capture ctDNA assay covering fusions in six relevant genes [6] or a cfTNA amplicon-based assay [16,17]. The challenges for comprehensive detection of actionable fusions and high value of RNA sequencing have already been emphasized on tissue [18]. As this type of molecular alterations has specific analytical challenges due to promiscuity of fusion partners and breakpoints, better characterization of concordance and sensibility of plasma-based assays is needed to ensure proper coverage of guideline-recommended genotyping in NSCLC.

Another interesting perspective related to this real-life evaluation of biomarker testing pertains to the advantage of liquid biopsy regarding delays. Indeed, several steps to complete the lung cancer biomarker testing trajectory can be replaced by a plasma-first approach, from the initial patient visit to the date when molecular results become available. As observed here, the 10 d average time for liquid biopsy results was inferior to the cumulative delays necessary to complete the biomarker testing from tissue. This is true even if TAT for both diagnosis and molecular testing on tissue, limited to baseline biomarkers (*EGFR*, *ALK*, *ROS1* and *PD-L1)*, were both within 3 d and the median cumulative TAT was 13 d. These short delays from our institution allow treatment decision planning to occur within 20 d in most cases. However, they might not be representative of general practice, as they result from optimized workflows [19] and are not estimating delays for sample shipping to a reference laboratory, per example. Nonetheless, this was achieved without relying on a more expensive and labor-intensive reflex-testing strategy advocated in similar public system practices [20,21].

Necessarily, the integration of tissue testing by NGS introduces longer delays for tissue genotyping trajectory as compared with minimal single-gene testing. This has the potential to further enhance the advantage of liquid biopsy on this aspect. In our laboratory, transition from single-gene testing to NGS resulted in a shift from 2.5 to 8 d (Patrice Desmeules, IUCPQ, Quebec, QC, Canada. Personal observation 2022.), per example, but delays above 15 days for tissue NGS are reported elsewhere, depending on the assay, volumetry and workflow used at the reference laboratory [13,15,17,22]. Not surprisingly, studies have documented reduced time to treatment using liquid biopsy as compared with tissue, especially if collected at visit before initiation of tissue biopsy [13,15,22]. In the present cohort, such comparison is not possible due to metachronous tissue and plasma testing, further limited by assays or approaches not covering all guideline-recommended biomarkers. Coverage of fusions would require more complex NGS strategy on plasma, translating potentially to longer TAT.

Another question not treated here regarding acceptance of liquid biopsy NGS for public health system governing authorities is its financial impact. Plasma NGS assays are still far more expensive than tissue NGS. As long as tissue biopsy remains necessary to complete standard of care *PD-L1* testing or complement the lower clinical sensitivity of liquid biopsy, procedural costs savings for acquiring tissue cannot be subtracted. A pragmatic integration of liquid biopsy testing into an algorithmic approach has been proposed by the IASLC committee [4], notably in the first line setting. The proposition is to use liquid biopsy either sequentially or complementarily if sub-optimal assay parameters or insufficient genotyping are obtained on tissue testing. The added value of complementary approaches has been previously demonstrated for patients in such scenarios and more limited tissue testing [9]. In addition, it could be proposed as a supplementary criterion that if the expected TAT for tissue genotyping is longer than 10 d or leads to a cumulative trajectory over 20 d, as per local service organization, a plasma-first approach could be defendable. In the context of searching for acquired resistance mechanism in oncogene-addicted cancers, the value of plasma testing is more evident but the capacity to capture fusions remains a key consideration as fusions are being increasingly recognized as resistance mechanisms to third-generation *EGFR*-inhibitors, notably [23,24]. Regardless of the scenarios to integrate liquid biopsy, the assay should capture all guideline-recommended biomarkers for NSCLC, thus including fusions.

## 5. Conclusions

In conclusion, the results from this retrospective study provide information about the added value of complementary plasma NGS genotyping as compared with minimal tissue testing with conventional methods from SGT. While additional molecular information was acquired in a large proportion of patients in a short TAT, the clinical sensitivity of plasma testing remains imperfect. Additionally, additional findings resulted only in a few patients undergoing a significant therapeutic change. This might be related to the regulatory context of the study population where access to emerging therapeutic agents is challenging and access to immunotherapy is widely adopted, and the fact that the plasma-based assay could not cover all guideline-recommended biomarkers, more specifically gene fusions and isoforms. As tissue NGS becomes more widely available and assuming it can be delivered within a clinically sensitive timeframe to cover all biomarkers in parallel to PD-L1, plasma-based NGS seems to be more appropriated as a complementary approach for patients with tumors insufficiently genotyped or inaccessible to tissue acquisition.

## Figures and Tables

**Table 1:** Baseline patients’ characteristics and biomarker outcomes.

Clinical and Pathological Characteristics	*N* (%)
Patients with liquid biopsy	91
Median age (range, y)	66 (27–83)
Sex	
Male	37 (41)
Female	54 (59)
Histology	
Adenocarcinoma	60 (66)
Non-small cell lung carcinoma, NOS	29 (32)
Squamous cell carcinoma	2 (2)
Stage at blood draw	
IIIB/C	4 (4)
IV	84 (92)
N/A	3 (3)
Site of metastatic disease	
Intra-thoracic	35 (38)
Extra-thoracic	51 (56)
Not available	5 (5)
Lines of treatment completed at time of liquid biopsy	
None	27 (30)
1	37 (41)
2 to 4	25 (27)
Not available	2 (2)
Clinical context at time of liquid biopsy	
Progression of disease	46 (51)
Diagnosis	23 (25)
Active therapy	13 (14)
Recurrent disease	9 (10)
Tissue biomarker testing performed by single-gene testing (SGT)	
EGFR/ALK	91 (100)
EGFR/ALK/PD-L1	88 (98)
EGFR/ALK/ROS1/PD-L1	76 (84)
EGFR/ALK/ROS1/BRAFV600/PD-L1	42 (46)
SGT + complementary NGS panel	13 (14)
Tissue biomarker result at blood draw by single-gene testing	
Driver Known and actionable	13 (14)
Driver Unknown	78 (86)

**Table 2:** Liquid biopsy SNV/indel detection rate per clinicopathological categories.

	Plasma Positive	Plasma Negative	Detection Rate (%)	Total (*n*) Evaluable	*p*-Value
All patients	43	48	47	91	
Tissue biopsy positive *	6	7	46	91	>0.9999
Tissue biopsy negative *	37	41	47		
Tissue PD-L1 > 50%	17	24	41	88	0.2930
Tissue PD-L1 50% or less	22	25	47		
No extra-thoracic spread	11	24	31	86	0.0278
Extra-thoracic spread	29	22	57		
On treatment	16	30	35	90	0.0340
Off treatment/naïve to treatment	26	18	59		

* By single-gene testing.

**Table 3:** Cases with potentially targetable driver alterations identified by liquid biopsy only.

Patient	Tissue Genotype	Plasma Finding	Therapy after BL Finding	Clinical Evolution
1	Negative	EGFR DEL19 *	Osimertinib	SD
2	Negative	ERBB2 INS20	Conventional	DOD
3	Negative	KRAS G12C	Sotorasib 2nd L	SD
4	Negative	KRAS G12C	Conventional	SD
5	Negative	KRAS G12C	Sotorasib 2nd L	Active treatment #
6	Negative	KRAS G12C	NA	NA
7	Negative	KRAS G12C	Sotorasib 2nd L	PD
8	Negative	KRAS G12C	Sotorasib 3rd L	Active treatment #
9	Negative	KRAS G12C	NA	NA
10	Negative	KRAS G12C	Conventional	SD
11	Negative	KRAS G12C	Conventional	PD

* Compound EGFR A750_E758del not covered by the PCR assay; L: line of therapy; SD: stable disease; PD: progressive disease; DOD: died of disease; NA: not available; #: not enough duration to evaluate radiologic response.

**Table 4:** Cases with potentially targetable driver alterations identified by tissue testing only.

Patient	Tissue Genotype	Plasma Finding	Therapy 1st L	Clinical Evolution
1	EGFR L861Q	Negative	Osimertinib	PR
2	EGFR L858R	Negative	Osimertinib	PR
3	EGFR DEL19	Negative	Osimertinib	PR
4	EGFR DEL19	Negative	Osimertinib	PD
5	EGFR DEL19	Negative	Osimertinib	PR
6	ALK fusion	Negative	Alectinib	SD
7	ALK fusion	Negative	Alectinib	PR

SD: stable disease; PD: progressive disease; PR: partial response; L: line of therapy.

**Table 5:** Summary of time-lapse for the main steps to obtain biomarker results in the cohort.

Delay Category; Days (Median); Range	Entire Cohort (*n* = 76)	Exclusion of Excessive Delays * (*n* = 61)	Liquid Biopsy (*n* = 91)
Procedures to acquire diagnostic material	7.9 (4); 1–44	6.8 (4); 1–35	–
Pathological diagnosis TAT	2.3 (2); 1–7	2.1 (2); 1–5	–
Pathological diagnosis to biomarker request	60.8 (2); 0–1133	3.0 (2); 2–24	–
Biomarker results TAT (single-gene testing)	2.8 (2); 2–7	2.8 (2); 2–7	–
Total trajectory for tissue testing	73.7 (13) 7–1137	14.7 (12); 7–64–	–
Liquid biopsy (blood draw to results)	–	–	10 (10); 6–25
*p*-value (tissue vs. liquid biopsy)	0.002	<0.0001	

* retrospective requests over 30 d and past resection specimens excluded from the main cohort.

## Data Availability

The data presented in this study are available on request from the corresponding author.
